# Oseltamivir Treatment for Influenza During the Flu Season of 2018–2019: A Longitudinal Study

**DOI:** 10.3389/fmicb.2022.865001

**Published:** 2022-05-10

**Authors:** Xiao-Guang Li, Jing Chen, Wei Wang, Fei Lin, Lu Li, Jing-Jin Liang, Zhong-Hua Deng, Bi-Ying Zhang, Ying Jia, Yuan-Bo Su, Yong-Feng Kang, Juan Du, Ya-Qiong Liu, Jie Xu, Qing-Bin Lu

**Affiliations:** ^1^Department of Infectious Diseases, Peking University Third Hospital, Beijing, China; ^2^Department of Laboratorial Science and Technology & Vaccine Research Center, School of Public Health, Peking University, Beijing, China

**Keywords:** oseltamivir, influenza virus, ILI, retrospective cohort study, clinical manifestation

## Abstract

**Background:**

Oseltamivir resistance in influenza virus (IFV) has been of widespread concern. An increase in the frequency of viruses with reduced inhibition was observed. Whether oseltamivir is effective is uncertain. We conducted this study to understand the real-world situation in northern China and the clinical efficacy for patients with IFV infection after the use of oseltamivir.

**Methods:**

The longitudinal study was performed on influenza-like illness (ILI) cases in a tertiary general hospital in Beijing, China during the flu season of 2018–2019. All ILI cases (≥18 years) were recruited into the study. We analyzed the effect of the oseltamivir therapy on the number of clinic visits, hospitalization frequency, and the duration of fever and cough.

**Results:**

A total of 689 ILI patients were recruited in this study with 355 in the oseltamivir therapy group and 334 in the supportive therapy group. Among the ILI patients, 388 patients were detected for IFV infection (364 IFV-A and 24 IFV-B) and divided into two groups with or without the oseltamivir therapy (302 vs. 86). There were no significant differences in the basic characteristics between the oseltamivir and supportive therapy groups in the ILI patients or in the IFV positive patients (all *p* < 0.05). After adjusting for the potential confounders, oseltamivir therapy reduced the times of clinic visits in the ILI and IFV positive patients (*p* = 0.043 and *p* = 0.011). No effectiveness with oseltamivir therapy was observed in the outcomes of hospitalization frequency, and the duration of fever and cough.

**Conclusion:**

Oseltamivir use may reduce the times of clinic visits. However, we did not observe the differences in the duration of fever, cough, and the frequency of hospitalization between oseltamivir therapy and supportive therapy.

## Introduction

Influenza is one of the important respiratory infections and is related to high morbidity and mortality in the community, which can cause about 290,000–650,000 deaths each year (Iuliano et al., 2018). Influenza vaccination and antiviral drugs are two important aspects for the prevention and treatment of influenza. In China, the population influenza vaccination rate is very low at only 2% ~ 3% per year (Yang et al., 2016). Therefore, antiviral drugs for influenza virus (IFV) appeared to be even more outstanding.

The first-line antiviral drugs worldwide are mainly oseltamivir and zanamivir. Oseltamivir (Tamiflu) was approved by the US Food and Drug Administration in 1999 for the treatment of uncomplicated influenza within 48 h of symptom onset. It was then widely used in various countries. In the worldwide pandemic of H1N1 influenza during 2009–2010, people were in panic and needed specific antiviral drugs. In 2010, oseltamivir was added to the WHO’s list of essential medications (World Health Organization, 2010). It is generally believed that oseltamivir is of great significance in reducing viral load, alleviating fever duration, shortening disease course, reducing flu-related complications, hospitalization rate, and mortality (Jefferson et al., 2014; Dobson et al., 2015). The latest US 2018 edition of the guidelines for the diagnosis and treatment of influenza also highlighted the status of oseltamivir as one of the single-drug options for starting treatment (Uyeki et al., 2019). In China, oseltamivir was not gradually used by medical personnel and patients until 2009. The availability of oseltamivir is also improving, and the policies and directions for its use are changing. In 2009, the Chinese Ministry of Health issued the guidelines that recommended the use of oseltamivir within 48 h of symptom onset, for high-risk and severe cases (Chinese Ministry of Health, 2009). In 2019, the indications of oseltamivir for medical insurance reimbursement in China also increased the number of people at a high risk of being diagnosed with influenza, which further promoted the use of drugs. However, it appears to show no benefit in starting treatment more than 48 h after symptom onset in hospitalized general medicine patients or outpatients infected with either H1N1 or other IFV strains or in doubling the dose of oseltamivir in hospitalized patients or outpatients (McQuade and Blair, 2015).

Oseltamivir resistance in IFV infection has been widely concerned. H274Y (N1), R292K (N2) neuraminidase mutations compromise viral fitness, which is confirmed to be related to the oseltamivir resistance (Bloom et al., 2010; Abed and Boivin, 2017; Hussain et al., 2017; Lackenby et al., 2018; Jia et al., 2019). These mutations can occur without drug pressure (Lackenby et al., 2018). WHO Collaborating Centers reported that the frequency of viruses with reduced inhibition has remained low since the year of 2012/13 (2015/16: 0.8%, 2014/15: 0.5%; 2013/14: 1.9%; 2012/13: 0.6%), and 2016/17 has the lowest frequency recorded at 0.2% (Lackenby et al., 2018). While the frequency of viruses with reduced inhibition was 3.3% in 2017 and 6.7% in 2018 in Guangdong Province, China. A randomized, double-blind, multicenter clinical trial reported that the median disease duration, the time to normal axillary temperature, normal living activities, and viral response were not significantly different among the peramivir group, the oseltamivir group, and the placebo group in patients with mild influenza (Fan et al., 2019). A multi-season cohort study reported that oseltamivir treatment had a better effect on severely ill patients with IFV A/H3N2 infection rather than on IFV A/H1N1 and B, which demonstrated that the efficacy of oseltamivir was not equal against all IFV types (Lytras et al., 2019). According to the weekly reports by the Chinese National Influenza Center, the IFV A/H1N1 and B were the predominant types during the 2018–2019 flu season. Whether the oseltamivir is effective is uncertain. Antiviral therapy within 48 h of symptom onset can reduce the complications, mortality, and hospitalization duration according to Diagnosis and Treatment of Influenza in China (National Health Commission of the People’s Republic of China, 2019). Therefore, it is necessary to evaluate the efficiency of oseltamivir in the early use to provide evidence for the use of oseltamivir in influenza-like illness (ILI) cases or IFV infected patients.

We conducted a longitudinal study on the ILI cases in a tertiary general hospital in Beijing, China during the flu season of 2018–2019 to understand the real-world situation in northern China and the clinical efficacy of patients with seasonal influenza after the use of oseltamivir.

## Materials and Methods

The longitudinal study was performed in the Peking University Third Hospital, Beijing during the flu season of 2018–2019 (December–March). All the ILI cases (≥18 years) were recruited for the study. The ILI cases were included according to the WHO definition as sudden onset fever (>38°C) with cough or sore throat, in the absence of other diagnoses.^1^ The excluded criteria were as follows: (1) the cases who visited the clinic after 2 days or more of symptom onset; (2) the cases used another antiviral drug other than oseltamivir; (3) the cases used oseltamivir for less than 5 days; and (4) the cases were lost to follow-up at the 30th day. The research protocol was approved by the ethics committee of Peking University Third Hospital. Written informed consent was obtained from all the patients.

The doctors from the fever clinic of the hospital surveyed and followed up the ILI cases. The basic information of the patients in the study collected by the doctors using a standardized questionnaire included age, sex, underlying diseases, status of smoking, the onset and clinical course of the infection (including the highest temperature, clinical manifestations, and laboratory tests), use of antiviral drugs (type of drugs and used days), the laboratory test results of IFV (A and B), the clinical manifestations at the 7th day after symptom onset, and clinical outcome (fever days, cough days, number of hospital visits and hospitalization). The colloidal gold method was applied immunochromatography and a double antibody sandwich to detect IFV-A/IFV-B antigens by IFV-A/IFV-B viral antigen detection kit (Guangzhou Wongfo Biotech Co., Ltd., Guangzhou, China) with the sensitivity and specificity of 73.8% and 66.3%, respectively (Li et al., 2021). The number of hospitalizations included the times in the Peking University Third Hospital and in other hospitals. The first follow-up was performed by telephone about 1 week after symptom onset. The second follow-up was performed about 4 weeks after symptom onset if the patient did not recover at the first follow-up.

The prescription of oseltamivir by doctors was mainly dependent on the diagnosis of influenza. If the influenza virus was positive and the imaging showed pneumonia, doctors would consider it as severe influenza and prescribe oseltamivir. If the patients belonged to a high-risk group for influenza, such as old age (more than 65 years), having underlying diseases, were pregnant, etc., oseltamivir would be prescribed. The wishes of the patient were also considered regarding the prescription of oseltamivir. The patients in the oseltamivir therapy group were administered oral oseltamivir 75 mg bid for 5 days and supportive therapy. The patients in the supportive therapy group only received the supportive therapy. The supportive therapy included rest, drinking more water, a bland diet, and taking Chinese patent medicine for sore throat and cough. The Chinese patent medicine mainly included the Ganmao Qingre granule, Feili cough mixture, and Lanqin oral liquid. Follow-up continued for about 4 weeks for all patients.

The primary outcome was fever days. We also examined cough days, the number of clinic visits, and hospitalization frequency as the secondary outcome. The people at high risk for flu complications were defined according to the Centers for Disease Control and Prevention.^2^ The number of clinic visit were classified into two groups of 1 and ≥2.

Descriptive statistics were performed for all variables; continuous variables were presented as means and SDs or as medians and interquartile ranges (IQRs), and categorical variables were presented as frequencies and proportions. An independent *t*-test, a *χ*^2^ test, a Fisher exact test, or a nonparametric test was used to determine the difference between the two groups where appropriate. The logistic regression model was used for calculating the effect of oseltamivir therapy on the number of clinic visits and the outcome of hospitalization; the generalized linear model was used for calculating the effect of oseltamivir therapy on the outcome of the duration of fever and cough. All the models were adjusted for the variables of age, sex, underlying disease, smoking status, high-risk population, white blood cell (WBC) group, mononuclear cell group, and neutrophils group. We also examined the effect of oseltamivir therapy on the outcome of the duration of fever and cough in the different groups using generalized linear models. The Kaplan–Meier method was used to analyze time-to-event data about the effect of oseltamivir therapy using the log-rank test; hazard ratios and 95% CIs were calculated based on a Cox regression model. A two-sided *p* < 0.05 was considered statistically significant. All analyses were performed using Stata 17.0 (Stata Corp LP, College Station, TX, United States).

## Results

### General Information

**Figure 1:**
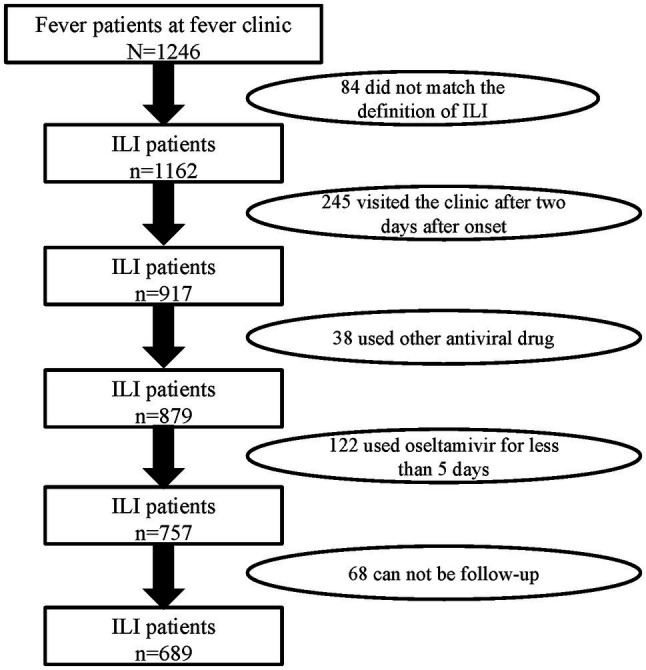
Flowchart of reported patients in the study population and reasons for exclusion.

There were 1,246 febrile patients at the fever clinic in the hospital during the flu season of 2018–2019. A total of 689 ILI patients meeting inclusion and exclusion criteria were recruited in this study (Figure 1). According to whether they were receiving oseltamivir therapy or not, the ILI patients were divided into two groups: the oseltamivir therapy group (355) and the supportive therapy group (334). The median age of the ILI patients was 31 years (IQR 24–39 years), the median of delayed days was 1 day (IQR 1–2 days), and 289 (41.9%) were men. Of these ILI patients, 59 (8.6%) had underlying diseases. About 6.4% (47/689) had a history of smoking, and 108 (15.7%) were at high risk for flu complications including being older than 65 or with underlying diseases or immunodeficiency. However, there were no significant differences in these characteristics between the two groups (*p* > 0.05; Table 1).

**Table 1:** The characteristics of the influenza-like illness (ILI) patients with or without oseltamivir therapy.

Characteristics	Total ILI patients (*N* = 689)	Oseltamivir therapy		*p*
		Yes (*n* = 355)	No (*n* = 334)	
Age, year, median (IQR)	31 (24–39)	31 (24–39)	30 (24–41)	0.887
<45	549 (79.7)	285 (80.3)	264 (79)	0.686
≥45	140 (20.3)	70 (19.7)	70 (21.0)	
Sex, male, *n* (%)	289 (41.9)	141 (39.7)	148 (44.3)	0.222
Delay, day, median (IQR)	1 (1–2)	1 (1–2)	1 (1–2)	0.442
Underlying diseases, *n* (%)	59 (8.6)	31 (8.7)	28 (8.4)	0.870
Hypertension	33 (4.8)	18 (5.1)	15 (4.5)	0.722
Coronary heart disease	11 (1.6)	7 (2.0)	4 (1.2)	0.548
Tumor	12 (1.7)	4 (1.1)	8 (2.4)	0.251
Diabetes	16 (2.3)	12 (3.4)	4 (1.2)	0.076
COPD	9 (1.3)	7 (2.0)	2 (0.6)	0.179
Cerebrovascular disease	3 (0.4)	1 (0.3)	2 (0.6)	0.613
Hepatitis/tuberculosis	3 (0.4)	0 (0)	3 (0.9)	0.113
Smoking	47 (6.8)	25 (7.0)	22 (6.6)	0.813
High risk people	108 (15.7)	59 (16.6)	49 (14.7)	0.482

IQR, interquartile range and COPD, chronic obstructive pulmonary disease.

Among the ILI patients, 388 patients were determined to be infected with IFV, including 364 (93.8%) IFV-A and 24 (6.2%) IFV-B. Their basic features were similar to the ILI patients. They also were divided into two groups with or without the oseltamivir therapy (302 vs. 86), and none of these features were significantly different (*p* > 0.05; Table 2).

**Table 2:** The characteristics of the patients infected with influenza virus with or without oseltamivir therapy.

Characteristics	Total patients infected with influenza virus (*n* = 388)	Oseltamivir therapy		*p*
		Yes (*n* = 302)	No (*n* = 86)	
Age, year, median (IQR)	31 (24–38)	31 (24–38)	30 (24–37)	0.912
<45	315 (81.2)	243 (80.5)	72 (83.7)	0.495
≥45	73 (18.8)	59 (19.5)	14 (16.3)	
Sex, male, *n* (%)	155 (40)	121 (40.1)	34 (39.5)	0.929
Delay, day, median (IQR)	1 (1–2)	1 (1–2)	1 (1–2)	0.584
Underlying diseases, *n* (%)	32 (8.3)	24 (8.0)	8 (9.3)	0.687
Hypertension	20 (5.2)	15 (5)	5 (5.8)	0.754
Coronary heart disease	7 (1.8)	5 (1.7)	2 (2.3)	0.653
Tumor	5 (1.3)	3 (1.0)	2 (2.3)	0.307
Diabetes	10 (2.6)	8 (2.7)	2 (2.3)	1.000
COPD	6 (1.6)	6 (2.0)	0 (0)	0.346
Cerebrovascular disease	1 (0.3)	0 (0)	1 (1.2)	0.222
Hepatitis/tuberculosis	1 (0.3)	0 (0)	1 (1.2)	0.222
Smoking	28 (7.2)	23 (7.6)	5 (5.8)	0.569
People at a high risk	62 (16.0)	47 (15.6)	15 (17.4)	0.675

IQR, interquartile range and COPD, chronic obstructive pulmonary disease.

### Comparison on Admission

Comparing the clinical symptoms and laboratory parameters on admission in the ILI patients with or without the oseltamivir therapy, frequently seen symptoms included pharyngalgia (67.9%), headache (69.6%), rhinorrhea (57.8%), and sputum (38.0%) in the oseltamivir therapy group, more than in the supportive therapy group (*p* < 0.05). The WBC level was lower, and the percentage of mononuclear cells was higher in the oseltamivir therapy group than in the supportive therapy group (*p* < 0.001), but patients with a lymphocyte percentage of less than 20% were more numerous among those treated with oseltamivir than with supportive treatment (*p* = 0.015). The median of the highest temperature in the oseltamivir therapy group was 38.7°C (IQR 38.3–39.1°C), which was higher than that in the supportive therapy group (*p* = 0.042). Other laboratory parameters in the two groups were not significantly different (Supplementary Table 1).

Among the patients infected with IFV, patients with the oseltamivir therapy on admission appeared higher frequencies of feeble (88.1%), pharyngalgia (68.2%), rhinorrhea (60.6%), and sputum (38%) than those without the oseltamivir therapy (*p* < 0.05). The median of the highest temperature in the oseltamivir therapy group was higher than that in the supportive therapy group (*p* < 0.05). However, the laboratory findings between the two groups were similar (Supplementary Table 2).

### Comparative Effectiveness

Following up on the clinical effect of the ILI and IFV positive patients, the patients with oseltamivir therapy were less likely to require two or more visits to the clinic than the supportive therapy patients (*p* < 0.05). There was no significant difference in the outcome of hospitalization, the duration of fever and cough, and the clinical symptoms on the seventh day from symptom onset (Table 3). After adjusting for the variables of age, sex, underlying diseases, smoking, high-risk population, WBC group, mononuclear cell group, and neutrophils group, oseltamivir therapy reduced the times of clinic visits in the ILI and IFV positive patients (*p* = 0.043 and *p* = 0.011). There was no effect of oseltamivir therapy on the outcome of hospitalization or duration of fever and cough (Table 4).

**Table 3:** Outcomes of the ILI and influenza virus-positive patients with or without oseltamivir therapy.

Outcome	ILI			Influenza virus positive		
	Oseltamivir therapy	Supportive therapy	*p*	Oseltamivir therapy	Supportive therapy	*p*
Times of clinic visit			0.042			0.014
1	323 (91.0)	287 (85.9)		278 (92.1)	71 (82.6)	
≥2	32 (9.0)	47 (14.1)		24 (7.9)	15 (17.4)	
Hospitalization	4 (1.1)	7 (2.1)	0.306	3 (1.0)	1 (1.2)	0.883
Duration of fever, days	2 (2–3)	2 (1–3)	0.570	2 (2–3)	2 (1–3)	0.380
1	82 (23.1)	85 (25.5)	0.084	71 (23.5)	22 (25.6)	0.296
2	134 (37.8)	131 (39.2)		115 (38.1)	38 (44.2)	
3	113 (31.8)	81 (24.3)		91 (30.1)	17 (19.8)	
≥4	26 (7.3)	37 (11.1)		25 (8.3)	9 (10.5)	
Duration of cough, days	6 (5–6)	6 (5–6)	0.828	6 (5–6)	6 (5–6)	0.908
<6	135 (38)	110 (33)	0.172	119 (39.4)	28 (32.6)	0.248
≥6	220 (62)	223 (67)		183 (60.6)	58 (67.4)	
**Clinical symptoms on the 7th day from disease onset**						
Diarrhea	0 (0)	0 (0)	1.00	0 (0)	0 (0)	1.000
Nausea	0 (0)	1 (0.3)	0.485	0 (0)	0 (0)	1.000
Vomiting	0 (0)	0 (0)	1.000	0 (0)	0 (0)	1.000
Dyspnea	1 (0.3)	0 (0)	1.000	1 (0.3)	0 (0)	1.000
Sputum	10 (2.8)	8 (2.4)	0.729	7 (2.3)	1 (1.2)	0.506
Chill	0 (0)	0 (0)	1.000	0 (0)	0 (0)	1.000
Rhinorrhea	12 (3.4)	5 (1.5)	0.111	11 (3.6)	2 (2.3)	0.741
Feeble	6 (1.7)	2 (0.6)	0.288	5 (1.7)	0 (0)	0.591
Headache	5 (1.4)	3 (0.9)	0.726	4 (1.3)	1 (1.2)	1.000
Muscle and joint pain	1 (0.3)	0 (0)	1.000	1 (0.3)	0 (0)	1.000
Cough	69 (19.4)	64 (19.2)	0.927	64 (21.2)	16 (18.6)	0.601
Pharyngalgia	11 (3.1)	8 (2.4)	0.573	10 (3.3)	2 (2.3)	1.000

ILI, influenza-like illness.

**Table 4:** Outcomes of the ILI and influenza virus-positive patients with or without oseltamivir therapy.

Outcome	ILI			Influenza virus positive		
	Adjusted β	95% CI	*p*	Adjusted β	95% CI	*p*
Times of clinic visit	−0.529	−1.040, −0.018	0.043	−0.949	−1.681, −0.218	0.011
Hospitalization	−0.177	−1.56, 1.206	0.802	0.459	−2.741, 3.659	0.779
Duration of fever	−0.056	−0.221, 0.109	0.507	0.073	−0.17, 0.315	0.557
Duration of cough	0.17	−0.253, 0.593	0.432	0.192	−0.424, 0.808	0.541

The binary logistic regression model was used for calculating the effect of oseltamivir therapy on the times of clinic visit and the outcome of hospitalization; the generalized linear model was used for calculating the effect of oseltamivir therapy on the outcome of the duration of fever and cough. All the models were adjusted for the variables of age, sex, underlying disease, smoke, high-risk population, WBC group, mononuclear cell group, and neutrophils group.

**Figure 2:**
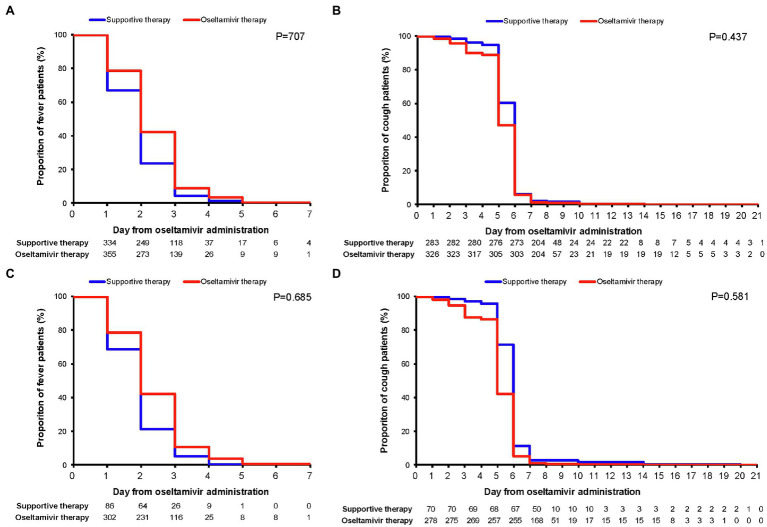
Kaplan–Meier plots for the remission of fever and cough. Comparisons (value of *p*) between the oseltamivir therapy and supportive therapy groups were performed with the log-rank test when adjusted for the variables of age, sex, underlying diseases, smoking, high-risk population, WBC group, mononuclear cell group, and neutrophils group. **(A)** Fever duration in ILI patients; **(B)** cough duration in ILI patients; **(C)** fever duration in influenza virus-positive patients; and **(D)** cough duration in influenza virus positive patients. WBC, white blood cell and ILI, influenza-like illness.

The Kaplan–Meier plots showed the remission of fever and cough in the oseltamivir therapy group was slower than that in the supportive therapy group when adjusted for the potential confounding variables. However, these were not significant by log-rank tests in the ILI patients or in the IFV positive patients (*p* > 0.05) in Figure 2. When classified by age, sex, underlying diseases, smoking, high-risk, white blood cell, neutrophils, and mononuclear cells, no differences were detected in the outcome of the duration days of fever and cough between the oseltamivir therapy and supportive therapy groups whether in the ILI patients or in the patients with IFV infection (Tables 5 and 6).

**Table 5:** Outcomes of the duration days of fever for the ILI patients with or without the oseltamivir therapy.

Characteristics	Duration of fever			Duration of cough		
	Adjusted β	95% CI	*p*	Adjusted β	95% CI	*p*
**Age**						
<45	−0.005	−0.186, 0.176	0.954	0.355	−0.106, 0.817	0.131
≥45	−0.180	−0.597, 0.237	0.397	−0.667	−1.706, 0.372	0.209
**Sex**						
Male	0.072	−0.178, 0.321	0.573	−0.067	−0.762, 0.628	0.850
Female	−0.154	−0.377, 0.069	0.176	0.371	−0.163, 0.905	0.173
**Underlying diseases**						
No	−0.072	−0.242, 0.099	0.411	0.220	−0.211, 0.651	0.317
Yes	0.160	−0.501, 0.821	0.635	−1.025	−2.892, 0.841	0.282
**Smoking**						
No	−0.060	−0.228, 0.107	0.479	0.201	−0.208, 0.609	0.335
Yes	0.155	−0.768, 1.078	0.742	0.059	−2.77, 2.888	0.967
**People at high risk**						
No	−0.043	−0.218, 0.133	0.632	0.213	−0.245, 0.672	0.362
Yes	−0.099	−0.582, 0.385	0.690	−0.166	−1.275, 0.942	0.769
**White blood cell**						
<10 × 10^9^/L	−0.098	−0.282, 0.085	0.293	0.171	−0.232, 0.574	0.406
≥10 × 10^9^/L	0.107	−0.339, 0.554	0.637	0.328	−1.294, 1.951	0.692
**Neutrophils, %**						
<70	0.056	−0.215, 0.328	0.683	−0.196	−0.83, 0.438	0.544
≥70	−0.112	−0.333, 0.108	0.318	0.384	−0.19, 0.957	0.190
**Mononuclear cell, %**						
<10	−0.132	−0.392, 0.129	0.321	0.013	−0.711, 0.737	0.972
≥10	−0.025	−0.262, 0.212	0.836	0.249	−0.311, 0.809	0.383

**Table 6:** Outcomes of the duration days of fever for the patients infected with influenza virus with or without oseltamivir therapy.

Characteristics	Duration of fever			Duration of cough		
	Adjusted β	95% CI	*p*	Adjusted β	95% CI	*p*
**Age**						
<45	0.077	−0.19, 0.344	0.572	0.324	−0.374, 1.021	0.363
≥45	0.038	−0.614, 0.69	0.910	−0.333	−1.6, 0.934	0.606
**Sex**						
Male	0.209	−0.181, 0.599	0.294	−0.767	−1.704, 0.169	0.108
Female	−0.050	−0.373, 0.272	0.759	0.901	0.049, 1.753	0.038
**Underlying diseases**						
No	0.052	−0.204, 0.309	0.689	0.256	−0.394, 0.905	0.441
Yes	0.152	−0.942, 1.247	0.785	−1.834	−4.374, 0.705	0.157
**Smoking**						
No	0.122	−0.119, 0.363	0.323	0.430	−0.18, 1.04	0.168
Yes	−0.920	−2.876, 1.035	0.356	−2.073	−6.101, 1.954	0.313
**People at high risk**						
No	0.089	−0.173, 0.351	0.506	0.189	−0.526, 0.905	0.605
Yes	−0.006	−0.722, 0.709	0.986	0.189	−0.746, 1.124	0.692
**White blood cell**						
<10 × 10^9^/L	0.096	−0.169, 0.362	0.477	0.369	−0.256, 0.994	0.247
≥10 × 10^9^/L	−0.046	−1.046, 0.955	0.929	−1.359	−4.832, 2.114	0.443
**Neutrophils, %**						
<70	0.240	−0.203, 0.683	0.288	0.175	−0.694, 1.045	0.692
≥70	0.020	−0.299, 0.338	0.904	0.084	−0.806, 0.974	0.853
**Mononuclear cell, %**						
<10	−0.053	−0.424, 0.318	0.779	−0.264	−1.478, 0.951	0.671
≥10	0.239	−0.134, 0.611	0.210	0.374	−0.386, 1.134	0.334

## Discussion

In the past decade, oseltamivir was one of the most important neuraminidase inhibitors in the treatment and prophylaxis of influenza. However, in our study, we did not find the efficiency of oseltamivir in the treatment of IFV during the flu season of 2018–2019 even if the oseltamivir use reduced the times of clinic visits.

In 2017, the WHO downgraded the status of oseltamivir from the core to the complementary list (Ebell, 2017), due to the evidence reducing the previously estimated magnitude of the effect of oseltamivir on relevant clinical outcomes in seasonal and pandemic flu. A meta-analysis published in 2013 showed only a 20-h mean reduction in symptoms and no evidence of a reduction in the likelihood of pneumonia, hospital admission, or complications requiring an antibiotic (Ebell et al., 2013). Freemantle et al. concluded that the findings on mortality, pregnancy, and neuropsychiatric events were interesting but inconclusive due to the small sample size and flawed study designs through doing the systematic review of observational studies about oseltamivir (Freemantle and Calvert, 2009; Freemantle et al., 2014). The evidence from the observational studies was weak and uncertain.

Dobson et al. (2015) performed a meta-analysis of randomized controlled trials including 9 trials with 4,328 patients, which reported that a 21% shorter time to alleviation of all symptoms for oseltamivir vs. placebo recipients was observed with 97.5 h for the oseltamivir group and 122.7 h for the placebo group. He et al. (2017) reported that the median duration of illness in the oseltamivir group was significantly shorter than that in the control group in 73 children identified as influenza-infected through laboratory tests while no significant differences were found in the median duration of illness or fever in 229 individuals with suspected influenza during 2015 in China. Different from the above studies, our study and the study by Fan et al. observed significant differences in symptom remission between the oseltamivir group and the placebo group neither in ILI patients nor in laboratory-confirmed IFV infection patients. This is consistent with the general trend of the WHO downgrading oseltamivir. In two studies, the patients were from the flu season of 2018–2019. The difference in the efficiency of oseltamivir may be caused by the changes in genetic sequences and genotypes, as well as the factors of population and environment.

IFV is continuously evolving through amino acid substitutions altering antigenic properties (antigenic drift) or, less frequently, by segment reassortment events (antigenic shift). Viral variants also form the occurrence and accumulation of neuraminidase resistance sites, increasing the risk of oseltamivir resistance. The effectiveness of oseltamivir in treating influenza was threatened by the predominance of oseltamivir resistance among seasonal H1N1 2009–2010, even in countries where oseltamivir had not been used (Meijer et al., 2009). However, the seasonal virus was subsequently displaced by the pH1N1 virus, which is largely sensitive to oseltamivir. It is noteworthy that oseltamivir-resistant strains were rarely reported during 2009–2010 (Lackenby et al., 2011). In the following years, a notable increase in the proportion of oseltamivir-resistant pH1N1 viruses was reported worldwide amongst patients with or without neuraminidase inhibitory activities (NAI) treatment (Storms et al., 2012; Hurt et al., 2016). This raises the concern that the prevalence of oseltamivir-resistant pH1N1 viruses may increase in the future as in the case of the previously circulating seasonal H1N1 viruses (Hurt et al., 2011; Takashita et al., 2014). Hebah et al. reported that epidemiological and genetic characterization of pH1N1 and H3N2 influenza viruses circulating in the Middle East and North Africa region during 2009–2017. Analysis of NA gene of pH1N1 viruses revealed sporadic detections of oseltamivir-resistance mutation, H275Y, in 4% of reported sequences, however, none of NAI resistance mutations were found in the NA of H3N2 viruses (Al Khatib et al., 2019). Lackenby et al. (2018) reported the frequency of viruses with reduced inhibition was the lowest frequency (0.5%) from 2,994 AH1N1 strains, 6,844 AH3N2 strains, and 2,242 B strains, which included 4 IFV-B strains from China. In Guangdong Province, the NAI resistance against A/H1N1 were detected in 2017 and 2018. IFV-B and A/H1N1 have been circulating during the flu seasons of 2017–2018 and 2018–2019 in China. Therefore, the current surveillance of influenza drug resistance in China may not be comprehensive enough, leaving out some hospitalized patients with severe illnesses, and may even be underestimated. The occurrence of oseltamivir-resistance against IFV-B and A/H1N1 may be related to the ineffective treatment. Of course, due to the low proportion of oseltamivir-resistant strains, it seems that drug resistance may play a minor role in the performance of ineffective treatment. No difference on the effectiveness between oseltamivir therapy and supportive therapy may be related to the highly reduced inhibition by either oseltamivir or zanamivir to IFV, as well as the occurrence of novel substitutions in NA (Lackenby et al., 2018; Price et al., 2020).

A meta-analysis showed that fewer admittances to hospital for any cause in the patients with oseltamivir therapy compared to those with the placebo (0.6% vs. 1.7%, RR 0.37, 95% CI 0.17–0.81; *p* = 0.013; Dobson et al., 2015). In our study, we found that the patients with supportive therapy had a higher frequency of clinic visits compared to the patients with oseltamivir therapy. This may be related to the psychological effects on patients. When the supportive therapy did not have an obvious effect, the patients were likely to see a doctor again. While the patients with the oseltamivir therapy probably had a strong psychological implication that the drug was effective, which may result in continuing taking medicine.

All data of the current study were highly reliable. However, the results and interpretations presented in the current study should be considered in the context of the following limitations. First, the sample size was not enough for sufficiently representative, especially for the laboratory-confirmed IFV infection patients with the supportive therapy, and the analysis did not differentiate the type of IFV and the possible interference of viral mutations and oseltamivir resistance was not considered, which may lead to selection bias. Second, the study excluded the patients who were cured within 5 days when administrated by oseltamivir, which may underestimate the effect of oseltamivir. Third, there was a recall bias for the reporting information by the patients during the follow-up investigation, which may bias the results. Four, the sensitivity and the specificity of the influenza antigen detection kit used in this study also affected the results. The low specificity might cause some false positive patients recruited in the study. The effectiveness of oseltamivir might be underestimated when the false positive patients were entered into the oseltamivir group.

The study evaluated the oseltamivir therapy on influenza during the flu season of 2018–2019. The oseltamivir use may reduce the times of clinic visits. However, we did not observe the differences in the duration of fever, cough, and the frequency of hospitalization between oseltamivir therapy and supportive therapy. Some studies also showed that oseltamivir in combination with antibiotics experienced a more rapid defervescence and a more rapid decline of IFV titer than the group treated with oseltamivir alone (Ishaqui et al., 2020; Lee et al., 2021). Due to the study design and sample size, further randomized controlled trials are needed to determine the efficacy of oseltamivir alone or in combination with antibiotics in treating influenza.

## Data Availability Statement

The raw data supporting the conclusions of this article will be made available by the authors, without undue reservation.

## Ethics Statement

The studies involving human participants were reviewed and approved by Peking University Third Hospital. The patients/participants provided their written informed consent to participate in this study.

## Author Contributions

X-GL, Q-BL, and JX designed the study and wrote the manuscript. JC, WW, FL, LL, J-JL, Z-HD, B-YZ, YJ, Y-BS, and Y-FK performed the experiment and collected the data. Q-BL, JD, Y-QL, and X-GL analyzed the data. All authors contributed to the article and approved the submitted version.

## Funding

The study was supported by the Clinical Key Project Youth Project in Peking University Third Hospital (BYSY2015017), National Major Science and Technology Project for the Control and Prevention of Major Infectious Diseases in China (2017ZX10103004), Haidian Prevention Medicine Association Funding (2017HDPMA04), and Peking University Medicine Seed Fund for Interdisciplinary Research (BMU2018MX009). The funding agencies had no role in the design and conduct of the study, collection, management, analysis, interpretation of the data, preparation, review, or approval of the manuscript.

## Conflict of Interest

The authors declare that the research was conducted in the absence of any commercial or financial relationships that could be construed as a potential conflict of interest.

## Publisher’s Note

All claims expressed in this article are solely those of the authors and do not necessarily represent those of their affiliated organizations, or those of the publisher, the editors and the reviewers. Any product that may be evaluated in this article, or claim that may be made by its manufacturer, is not guaranteed or endorsed by the publisher.

## Footnotes

^1^ https://www.who.int/influenza/surveillance_monitoring/ili_sari_surveillance_case_definition/en/

^2^ https://www.cdc.gov/flu/highrisk/index.htm?CDC_AA_refVal=https%3A%2F%2Fwww.cdc.gov%2Fflu%2Fabout%2Fdisease%2Fhigh_risk.htm

## Supplementary Material

Supplementary Table 1 Clinical manifestation and laboratory parameters on admission in the influenza-like illness patients with or without oseltamivir therapy.

Supplementary Table 2 Clinical manifestation and laboratory parameters on admission in the patients infected with influenza virus with or without oseltamivir therapy.
