# Supplementary material for: Oseltamivir Treatment for Influenza During the Flu Season of 2018–2019: A Longitudinal Study
Source: Front Microbiol. 2022 May 10;13:865001. doi: 10.3389/fmicb.2022.865001 (PMC9127596; doi:10.3389/fmicb.2022.865001)
Supplement: Supplementary Table 1 — Clinical manifestation and laboratory parameters on admission in the influenza-like illness patients with or without oseltamivir therapy. [file Table_1.docx]

Supplemental Table 1. Clinical manifestation and laboratory parameters on admission in the influenza-like illness patients with or without the oseltamivir therapy

| Laboratory test | ILI patients | Oseltamivir therapy | | P |
| --- | --- | --- | --- | --- |
|  |  | Yes | No |  |
| Cough | 589 (85.5) | 302 (85.1) | 287 (85.9) | 0.749 |
| Feeble | 585 (84.9) | 309 (87) | 276 (82.6) | 0.106 |
| Muscle and joint pain | 535 (77.7) | 284 (80) | 251 (75.2) | 0.127 |
| Pharyngalgia | 443 (64.3) | 241 (67.9) | 202 (60.5) | 0.043 |
| Headache | 440 (63.9) | 247 (69.6) | 193 (57.8) | 0.001 |
| Rhinorrhea | 330 (47.9) | 205 (57.8) | 125 (37.4) | <0.001 |
| Sputum | 228 (33.1) | 135 (38.0) | 93 (27.8) | 0.005 |
| Chill | 209 (30.3) | 110 (31) | 99 (29.6) | 0.701 |
| Dyspnea* | 118 (17.1) | 62 (17.5) | 56 (16.8) | 0.808 |
| Vomiting | 105 (15.2) | 59 (16.6) | 46 (13.8) | 0.299 |
| Nausea* | 35 (5.1) | 13 (3.7) | 22 (6.6) | 0.081 |
| Diarrhea | 11 (1.6) | 2 (0.6) | 9 (2.7) | 0.032 |
| White blood cell, ×10^9^/L, median (IQR) | 6.75 (5.40-8.68) | 6.41 (5.22-7.94) | 7.61 (5.79-9.78) | <0.001 |
| <10×10^9^/L | 786 (85.4) | 497 (91.2) | 289 (77.1) | <0.001 |
| ≥10×10^9^/L | 134 (14.6) | 48 (8.8) | 86 (22.9) |  |
| Hemoglobin, g/L, mean±SD | 144 (132-156) | 144 (132-155) | 145 (131-157) | 0.402 |
| Platelet counts, ×10^9^/L, median (IQR) | 186 (159-219) | 183 (158.5-215) | 190 (161-224) | 0.082 |
| Percentage of neutrophils, %, median (IQR) | 72 (63.8-78.8) | 70.7 (63.2-77.35) | 73.55 (64.6-80.9) | 0.617 |
| Percentage of lymphocytes, %, median (IQR) | 15.2 (10-20.75) | 15.9 (10.8-21.25) | 13.95 (9-19.5) | 0.125 |
| <20% | 639 (72) | 361 (68.9) | 278 (76.4) | 0.015 |
| ≥20% | 249 (28) | 163 (31.1) | 86 (23.6) |  |
| Percentage of mononuclear cell, %, median (IQR) | 10.7 (7.9-13.3) | 11.3 (8.8-13.8) | 9.8 (6.7-12.3) | <0.001 |
| <10% | 381 (42.9) | 195 (37) | 186 (51.4) | <0.001 |
| ≥10% | 508 (57.1) | 332 (63) | 176 (48.6) |  |
| Highest temperature, ℃, median (IQR) | 38.7 (38.3-39.0) | 38.7 (38.3-39.1) | 38.6 (38.2-39.0) | 0.042 |

IQR, interquartile range; SD, standard deviation; ILI, influenza-like illness.
