# Supplementary material for: Oseltamivir Treatment for Influenza During the Flu Season of 2018–2019: A Longitudinal Study
Source: Front Microbiol. 2022 May 10;13:865001. doi: 10.3389/fmicb.2022.865001 (PMC9127596; doi:10.3389/fmicb.2022.865001)
Supplement: Supplementary Table 2 — Clinical manifestation and laboratory parameters on admission in the patients infected with influenza virus with or without oseltamivir therapy. [file Table_2.docx]

Supplemental Table 2. Clinical manifestation and laboratory parameters on admission in the patients infected with influenza virus with or without the oseltamivir therapy

| Variables | Influenza virus positive cases | Oseltamivir therapy | | P |
| --- | --- | --- | --- | --- |
|  |  | Yes | No |  |
| Cough | 302 (77.8) | 256 (84.8) | 71 (82.6) | 0.619 |
| Feeble | 334 (86.1) | 266 (88.1) | 68 (79.1) | 0.033 |
| Muscle and joint pain | 305 (78.6) | 242 (80.1) | 63 (73.3) | 0.170 |
| Headache | 271 (69.9) | 218 (72.2) | 53 (61.6) | 0.060 |
| Pharyngalgia | 248 (63.9) | 206 (68.2) | 42 (48.8) | 0.001 |
| Rhinorrhea | 220 (56.7) | 183 (60.6) | 37 (43) | 0.004 |
| Sputum | 146 (37.6) | 119 (39.4) | 27 (31.4) | 0.176 |
| Chill | 119 (30.7) | 96 (31.8) | 23 (26.7) | 0.371 |
| Dyspnea | 68 (17.5) | 57 (18.9) | 11 (12.8) | 0.331 |
| Vomiting | 65 (16.8) | 53 (17.6) | 12 (14.0) | 0.431 |
| Nausea | 16 (4.1) | 11 (3.6) | 5 (5.8) | 0.372 |
| Diarrhea | 2 (0.5) | 2 (0.7) | 0 (0) | 1.000 |
| White blood cell, ×10^9^/L, median (IQR) | 6.69 (5.48-8.37) | 6.64 (5.46-8.27) | 6.95 (5.72-8.66) | 0.132 |
| <10×10^9^/L | 335 (89.3) | 267 (90.2) | 68 (86.1) | 0.291 |
| ≥10×10^9^/L | 40 (10.7) | 29 (9.8) | 11 (13.9) |  |
| Hemoglobin, g/L, mean±SD | 143 (131-153) | 144 (133-154) | 140 (127-153) | 0.259 |
| Platelet counts, ×10^9^/L, median (IQR) | 188 (164-220) | 187 (164-216) | 191 (163-227) | 0.732 |
| Percentage of neutrophils, %, median (IQR) | 72.6 (65.4-78.9) | 71.6 (64.7-78.2) | 74.7 (68.8-80.9) | 0.443 |
| Percentage of lymphocytes, %, median (IQR) | 15.1 (10-20.1) | 15.5 (10.4-20.6) | 13.1 (8.8-18) | 0.806 |
| <20% | 266 (74.1) | 207 (71.9) | 59 (83.1) | 0.053 |
| ≥20% | 93 (25.9) | 81 (28.1) | 12 (16.9) |  |
| Percentage of mononuclear cell, %, median (IQR) | 11 (8.4-13.8) | 11.3 (8.8-14) | 10.2 (7.4-12.1) | 0.995 |
| <10% | 142 (39.6) | 107 (37.4) | 35 (48.0) | 0.100 |
| ≥10% | 217 (60.5) | 179 (62.6) | 38 (52.1) |  |
| Highest temperature, ℃, median (IQR) | 38.7 (38.3-39.1) | 38.8 (38.3-39.2) | 38.5 (38.2-39) | 0.014 |

IQR, interquartile range; SD, standard deviation.
